# A Novel pAA Virulence Plasmid Encoding Toxins and Two Distinct Variants of the Fimbriae of Enteroaggregative *Escherichia coli*

**DOI:** 10.3389/fmicb.2017.00263

**Published:** 2017-02-22

**Authors:** Rie Jønsson, Carsten Struve, Erik J. Boll, Nadia Boisen, Katrine G. Joensen, Camilla A. Sørensen, Betina H. Jensen, Flemming Scheutz, Håvard Jenssen, Karen A. Krogfelt

**Affiliations:** ^1^Department of Science and Environment, Roskilde UniversityRoskilde, Denmark; ^2^Department of Microbiology and Infection Control, Statens Serum InstitutCopenhagen, Denmark

**Keywords:** enteroaggregative *E. coli*, EAEC, diarrhea, virulence genes, fimbriae

## Abstract

Enteroaggregative *Escherichia coli* (EAEC) is an increasingly recognized pathogen associated with acute and persistent diarrhea worldwide. While EAEC strains are considered highly heterogeneous, aggregative adherence fimbriae (AAFs) are thought to play a pivotal role in pathogenicity by facilitating adherence to the intestinal mucosa. In this study, we optimized an existing multiplex PCR to target all known AAF variants, which are distinguished by differences in their pilin subunits. We applied the assay on a collection of 162 clinical Danish EAEC strains and interestingly found six, by SNP analysis phylogenetically distinct, strains harboring the major pilin subunits from both AAF/III and AAF/V. Whole-genome and plasmid sequencing revealed that in these six strains the *agg3A* and *agg5A* genes were located on a novel pAA plasmid variant. Moreover, the plasmid also encoded several other virulence genes including some not previously found on pAA plasmids. Thus, this plasmid endows the host strains with a remarkably high number of EAEC associated virulence genes hereby likely promoting strain pathogenicity.

## Introduction

Enteroaggregative *Escherichia coli* (EAEC) is a common diarrheal organism and has been associated with acute and persistent diarrhea in a variety of settings (Cravioto et al., 1991; Cobeljic et al., 1996; Huppertz et al., 1997; Harrington et al., 2006; Scavia et al., 2008; Hebbelstrup Jensen et al., 2014). However, EAEC strains express a heterogeneous array of putative virulence factors, therefore, recognition of specific pathogenic factors within this pathotype remains challenging (Nataro et al., 1995a; Boisen et al., 2012; Estrada-Garcia and Navarro-Garcia, 2012; Lima et al., 2013).

Enteroaggregative *E. coli* was first defined by its distinctive “stacked-brick” pattern of aggregative adherence (AA) to HEp-2 cells, mediated by the aggregative adherence fimbriae (AAFs; Nataro et al., 1992). AAFs are believed to play a key role in EAEC pathogenicity by promoting host intestinal colonization via binding to the host intestinal mucosa (Yamamoto et al., 1991; Hicks et al., 1996).

The AAFs have been shown to promote biofilm formation and adhesion to various surfaces and are associated with inflammatory responses in the host (Sheikh et al., 2001; Harrington et al., 2005; Boisen et al., 2008; Farfan et al., 2008; Boll et al., 2012, 2013). Currently, five allelic variants are described (AAF/I-AAF/V), all sharing high level of conservation among the accessory genes whereas the pilin genes display much greater divergence (Savarino et al., 1994; Czeczulin et al., 1997; Bernier et al., 2002; Boisen et al., 2008; Jonsson et al., 2015; Nagy et al., 2016). The AAFs belong to the chaperone-usher family of adhesins, and consist of the pore-forming usher (encoded by *agg3C* in AAF/III), the chaperone (encoded by *agg3D* in AAF/III), the long fimbriae which consist of polymerized major pilin subunits (encoded by *agg3A* in AAF/III) and a minor pilin subunit at the tip (encoded by *agg3B* in AAF/III; Savarino et al., 1994; Berry et al., 2014).

The newest variant, AAF/V, was discovered just recently, and was revealed to be a chimeric variant of AAF/III, in which all accessory genes (*agg3CD*) as well as the minor pilin subunit gene (*agg3B*) are shared except of the major pilin subunit gene that is replaced by *agg5A* (Jonsson et al., 2015).

The expression of AAFs is regulated by an AraC-like transcriptional activator called AggR, which is shown to be an important virulence regulator in EAEC (Nataro et al., 1994; Morin et al., 2010). Most of the virulence genes of EAEC, including AggR, the surface protein dispersin and the AAFs, are located on a 72–120 kb plasmid, termed the pAA plasmid (Nataro et al., 1992; Johnson and Nolan, 2009). In addition to regulating the virulence factors on the plasmid, AggR also promote the expression of the AggR Activated Island (Aai), a type VI secretion system, encoded by a pathogenicity island (designated PAI-1) inserted at *pheU* on the chromosome in EAEC prototype strain 042 (Morin et al., 2013).

Currently, five pAA plasmids have been completely sequenced, each harboring the genes encoding for one of the five respective AAF variants. Although the pAA plasmids share common features including EAEC-associated virulence genes, the genetic composition and the synteny of the plasmids are quite different. Some of the *E. coli* pAA plasmids encode toxins, such as the autotransporter Pet and EAST1 in strain 042, SepA in the German O104:H4 outbreak strain C227-11, and EAST1 in strain 55989 (Eslava et al., 1998; Rasko et al., 2011).

Since AAF mediated adhesion is believed to play an essential role in EAEC pathogenicity, a better understanding of the prevalence of the different AAF variants and their relation to clinical outcome will contribute to the overall understanding of this important pathogen and potentially pave the way for future treatment strategies in the form of anti-adhesion therapy.

In this study, we optimized a previously described AAF multiplex PCR assay (Boisen et al., 2012) to include the recently identified AAF variant AAF/V and investigated the distribution of AAF variants in a collection of 162 clinical EAEC isolates. Interestingly, six isolates were found to harbor two AAF variants and these were revealed to be encoded by a novel pAA variant also encoding several other virulence factors, suggesting a key role of this plasmid in EAEC pathogenicity.

## Materials and Methods

### Bacterial Strains

*Escherichia coli* strains were isolated from stool specimens of adults suffering from diarrhea, during a multicenter study with participants: the Departments of Clinical Microbiology at Hvidovre Hospital (HH) and Slagelse Hospital (SH), and the Department of Microbiology and Infection Control at Statens Serum Institut (SSI). EAEC strains were identified from the genes *aatA, aggR, aaiC* and *aap* targeted by multiplex PCR as previously described (Boisen et al., 2012). Patients with EAEC positive stool samples, without any co-infections were eligible for inclusion in this study. The bacterial strains used in this study are described in **Table 1**. Stock cultures were frozen at -80°C in Luria-Broth (LB) with 10% glycerol. All strains were grown at 37°C.

**Table 1:** Strains used in this study.

Strains	Description	Reference
55989	Prototype EAEC strain expressing AAF/III	Bernier et al., 2002
C338-14	Prototype EAEC strain expressing AAF/V	Jonsson et al., 2015
C700-09	EAEC strain carrying *agg3A* and *agg5A* simultaneously	Boisen et al., 2012
C763-16	EAEC strain carrying *agg3A* and *agg5A* simultaneously	This study
C761-16	EAEC strain carrying *agg3A* and *agg5A* simultaneously	This study
C760-16	EAEC strain carrying *agg3A* and *agg5A* simultaneously	This study
C762-16	EAEC strain carrying *agg3A* and *agg5A* simultaneously	This study
C764-16	EAEC strain carrying *agg3A* and *agg5A* simultaneously	This study

### PCR

DNA templates for the multiplex PCR were prepared by boiling a suspension of five isolated colonies in 200 μL mQ water. The AAF multiplex was optimized from a previous study conducted by Boisen et al. (2012). The primers for the multiplex are listed in **Table 2**. The PCR was performed with the multiplex PCR kit according to the manufacturer’s instructions (Qiagen inc., Valencia, CA, USA). The multiplex PCR cycles comprised (a) 15 min denaturation at 95°C, (b) 30 s denaturation at 94°C, (c) annealing for 1.5 min, and (d) extension 1.5 min at 72°C with 35 cycles from step (b). The final extension was 10 min at 72°C.

**Table 2:** Primers used in this study.

	*Gene*	Description	Primer sequence	Amplicon size (bp)	Annealing temperature
*Optimized AAF multiplex*	*agg4A*	Major pilin subunit of AAF/IV	F 5′-TGAGTTGTGGGGCTAYCTGGA-′3 R 5′-CACCATAAGCCGCCAAATAAGC-′3	169	57
	*aggA*	Major pilin subunit of AAF/I	F 5′-TCTATCTRGGGGGGCTAACG-′3 R 5′-ACCTGTTCCCCATAACCAGAC-′3	218	
	*aafA*	Major pilin subunit of AAF/II	F 5′-CTACTTTATTATCAAGTGGAGCCGCTA-′3 R 5′-TAGGAGAGGCCAGAGTGWATCC-′3	292	
	*agg3A*	Major pilin subunit of AAF/III	F 5′-AGCTAGTGCTACTGCAAAATTAAAGTT-′3 R 5′-CAGGTTTAATATATTGGTCTGGAATAAC–′3	359	
	*agg5A*	Major pilin subunit of AAF/V	F 5′-GTTTCATCAACTGGAACTATTACTATTT–′3 R 5′-TAATTTAAGCTGAAGAATCCAGTCAAT-′3	401	
	*agg3/4C*	Usher-encoding protein of three AAF variants; III, IV and V	F 5′-CATARTGAAGGTATAACATTTGGTCAGA-′3 R1 5′-GTCAGCATAACACTTACTGTTCATTC-′3 R2 5′-GTAGTTTGCATAGCAATGGCTATTCATT-′3	477	
	*rpoA*	qRT-PCR	5′-GAAGAGATGGATGACGACGAAGACG-′3 5′-GTACGCAGCTCGGCGAATTTCTCAC-3′	115	60
	*Agg3A*	qRT-PCR	F 5′-GCGTGGGAACAAATACTGGACA-′3 R 5′-TGGTCTGGAATAACAGCTTGAACTC-′3	81	60
	*Agg5A*	qRT-PCR	F 5′-TTTCCATCTTTAGCATCTGCCC-′3 R 5′-ATTAGTTCGCCTCCTCCTGT-′3	145	60

### Plasmid Profiling

Plasmids were purified according to the method descried by Kado and Liu (1981), followed by separation on a 0.8% (w/v) agarose gel and stained with GelRed^®^ (Biotium, Hayward, CA, USA). The approximate molecular weight of each plasmids were determined by comparing with the reference *E. coli* strain 39R861, harboring four plasmids of 6.9-, 36-, 63- and 147 kb (Macrina et al., 1978).

### MinION Sequencing of the Plasmids of C700-09

The plasmid DNA of C700-09 was extracted using QIAGEN Plasmid Midi kit (QIAGEN, Copenhagen, Denmark) according to the manufacturer’s instructions. The plasmids were sequenced commercially using the MinION sequencing platform (DNASense, Aalborg, Denmark). The fully assembled pAA^C700-09^ is deposited in the EMBL-EBI repository^1^ under project number PRJEB18579.

### Whole-Genome-Sequencing

Genomic DNA was extracted from isolates using the DNeasy blood & tissue kit (QIAGEN, Copenhagen, Denmark) and fragment libraries were constructed using a NexteraTM kit (Illumina, Little Chesterford, UK) followed by 251-bp paired end sequencing (MiSeq; Illumina) according to the manufacturer’s instructions. Reads were assembled *de novo* using CLC GenomicsWorkbench 7.5 (QIAGEN, Aarhus, Denmark). The whole-genome sequencing data is deposited in the EMBL-EBI repository^2^ under the primary identification number PRJEB18579.

### Sequence Analysis

The pAA plasmid was annotated using the RAST annotating system (Aziz et al., 2008). Putative hypothetical genes identified by RAST annotation were manually curated using NCBI BLASTn and BLASTp searches. Putative EAEC genes were mapped to a panel of EAEC plasmids/genomes using CLC Main Workbench 7.5.1. BLASTn atlases of the pAA virulence plasmids were constructed using BLAST Ring Image Generator v0.95 (BRIG; Alikhan et al., 2011).

### Serotyping

Serotyping was performed in silico by using serotype finder^3^. Phenotypical serotyping was performed at the International *Escherichia* and *Klebsiella* Centre (World Health Organization), Department of Microbiology and Infection Control, Statens Serum Institut, Copenhagen, Denmark using methods described elsewhere (Orskov and Orskov, 1992).

### RNA Extraction and qRT-PCR

For optimal expression of the AAFs, overnight cultures grown in LB-broth were diluted 1:100 in 20 ml Dulbecco’s modified Eagle Medium (DMEM) supplemented with 0.45% glucose (DMEM-HG). The strains were incubated with shaking at 37°C until an optical density (OD_600_) of 0.8. RNA was extracted with the RNase mini kit with the addition of an on-column digestion in order to remove contaminating DNA using the RNAse-Free DNase Set (Qiagen, Inc, Valencia, CA, USA). The RNA was quantified by Qbit analysis and cDNA was synthesized from 1 μg of bacterial RNA using random hexamer primers and the Thermoscript reverse transcriptase (RT) enzyme (Invitrogen, Carlsbad, CA, USA) for 10 min at 25°C, 1 h at 50°C, and 5 min at 85°C. As negative controls, all samples were tested without the addition of RT. The RNA was extracted from three independent experiments, and the qRT-PCR reactions were performed in duplicates on each sample. qRT-PCR was performed by using a 7500 real-time PCR system (Applied Biosystems, Foster City, CA, USA) and the expression level for each queried gene was normalized to the constitutively expressed *rpoA* gene as previously described (Fujimitsu et al., 2008).

### Statistical Analyses

Data were analyzed by performing two-tailed or, when appropriate, one-tailed Mann–Whitney tests using GraphPad Prism 6 software (GraphPad Software, San Diego, CA, USA). Each value used for statistical analyses is the mean of three replicates. Results were considered significant when *P* < 0.05 (^∗^), *P* < 0.01 (^∗∗^), *P* < 0.001 (^∗∗∗^).

## Results

### Optimization of an AAF PCR Multiplex Assay to Include AAF/V

**FIGURE 1:**
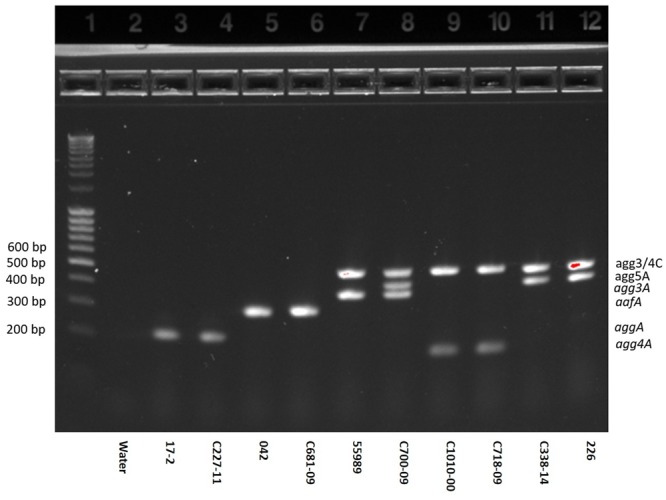
**Agarose gel of the optimized AAF multiplex demonstrated on 10 EAEC strains.** PCR products representing genes for *aggA* (AAF/I)*, aafA* (AAF/II)*, agg3/4C* (usher of AAF/III, AAF/IV and V, all detected by the same primer set)*, agg3A* (AAF/III)*, agg4A* (AAF/IV) and *agg5A* (AAF/V) were separated on a 1.2% agarose gel. Lane 1: ladder, lane 2: negative control (water), lanes 3–4: EAEC strains 17-2 and C227-11 both harboring *aggA*, lanes 5–6: EAEC strains 042 and C681-09 both harboring *aafA*, lane 7: EAEC strain 55989 harboring *agg3A* and *agg3C*, lane 8: EAEC strain C700-09 harboring *agg3A, agg3C* and *agg5A*, lanes 9–10: EAEC strains C1010-00 and C718-09 both harboring *agg4A* and *agg4C*, lanes 11–12: EAEC strains C338-14 and 226 both harboring *agg5A* and *agg3C*. As shown in lane 8, the strain C700-09 tested positive for the usher of *agg3C* as well as the *agg3A* and *agg5A* gene.

A novel AAF variant, AAF/V, was recently identified. We here optimized a previously described multiplex PCR assay to detect AAF variants (Boisen et al., 2012) also to include AAF/V. Whereas, the previously described multiplex detected several of the usher genes from AAF/I-IV, we decided only to include the genes encoding the five major pilin subunits and the usher found in AAF/III, AAF/IV or AAF/V (*agg3/4C*) which can be detected by a single primer set. The multiplex PCR was validated by testing EAEC strains, with known AAF profiles. All strains exhibited the expected AAF gene profiles. However, surprisingly, we found that one strain (C700-09) harbored two distinct fimbrial genes; not only *agg3A* but also the gene encoding *agg5A* (**Figure 1**, Lane 8), along with the usher encoding gene (*agg3/4C*).

### Prevalence of AAFs in a Collection of Clinical EAEC Isolates

We next used the optimized PCR multiplex assay to investigate the prevalence of the different AAF variants in a collection of 162 EAEC isolates isolated from Danish patients suffering from diarrhea. The most frequent gene encoding an AAF major pilin subunit was *aggA* (25.3%) encoding the AAF/I major pilin subunit, followed by *agg4A* (17.3%; AAF/IV), *agg5A* (14.2%; AAF/V), *agg3A* (13.6%; AAF/III) and *aafA* at 13.6% (AAF/II). Thus, there was a high degree of resemblance of the prevalence of the AAF variants to previous studies (Boisen et al., 2012; Lima et al., 2013; Jonsson et al., 2015). Twenty-one strains (13%) were negative for all five AAF variants.

Surprisingly, as observed for isolate C700-09 above, five (3.1%) of the tested strains were positive for two major pilin genes, *agg3A* and *agg5A* encoding the major pilin subunit of AAF/III and AAF/V, respectively.

### Identification of a Novel pAA Plasmid Encoding Both AAF/III and AAF/V Genes

To further investigate the EAEC strains harboring two distinct AAF variants, we isolated plasmids from strain C700-09 and sequenced them using the Oxford Nanopore MinION technology. C700-09 was found to harbor three plasmids of 5.4, 72, and 120 kb, respectively. By BLAST analysis, the 120 kb plasmid was identified as the pAA plasmid (pAA^C700-09^), whereas the 72 kb plasmid was found to be a resistance plasmid encoding antibiotic resistance genes [*strA, strB, blaTEM-1B, sul2, tet(B), dfrA8*]. The smallest plasmid of 5.4 kb encoded 11 hypothetical proteins.

**FIGURE 2:**
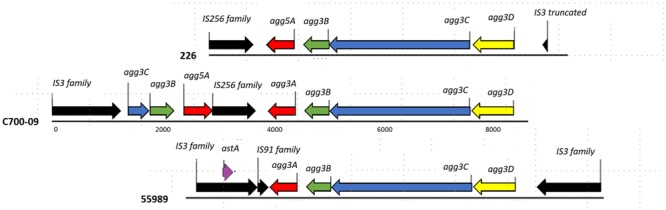
**Comparison of the genetic organization of the AAF cluster.** Annotation of the chimeric cluster of C700-09 compared to the two AAF clusters (AAF/III from strain 55989 and AAF/V from strain 226). The genes encoding the assembly genes (*agg3DC*) as well as the minor pilin subunit (*agg3B*) are shared between AAF/III and AAF/V as previously described (Jonsson et al., 2015). Different colors represent different gene functions: black, mobile elements; purple, the gene encoding EAST-1; yellow, the chaperone-encoding gene; blue, the usher-encoding gene; green, the minor pilin subunit; and red, the major pilin subunit. IS elements are named according to ISfinder and the numbers below the lines corresponds to basepairs.

The sequencing data revealed that the pAA plasmid contained a region encoding the AAF/III-gene cluster followed by a truncated variant of the AAF/V cluster encoded by the opposite strand (**Figure 2**). The two AAF gene clusters were interspaced by a 1025 bp region encoding an insertion element. The truncated AAF/V gene-cluster lacked the entire *agg3D* gene and the majority of the *agg3C* gene was replaced by an insertion element (**Figure 2**). In addition to the fimbriae-encoding region, the pAA plasmid included 186 putative ORFs of which 50 encoded for hypothetical proteins with no match in the RAST database. Twelve putative ORFs were involved in catabolism and metabolism, four putative ORFs encoded for transmembrane proteins and 15 were involved in transfer, replication, or plasmid maintenance function. Sixty-two putative ORFs were mobile elements including integrases, transposases or phage related factors and the remaining 43 putative ORFs have been demonstrated or predicted to have a role in virulence.

**FIGURE 3:**
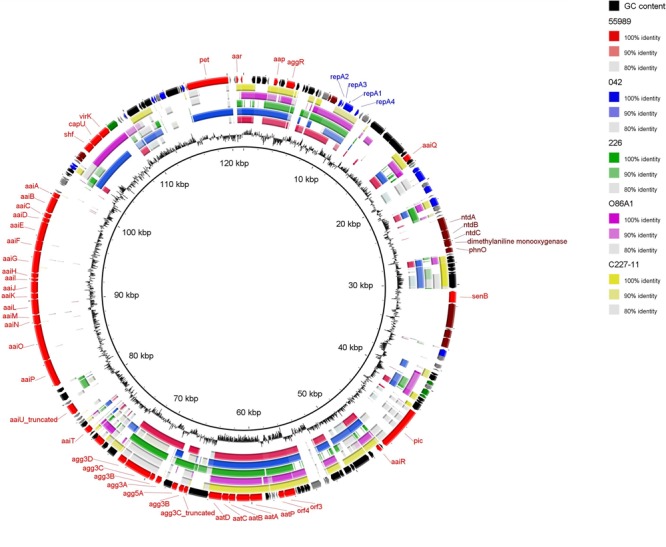
**Circular map of pAA^C700-09^ compared to the other public available pAA plasmids each harboring a different AAF variant.** The outer ring shows predicted ORFs. Different colors represent different putative functions: gray, hypothetical proteins; red, EAEC-specific virulence factors; blue, plasmid replication and maintenance; maroon, catabolism and metabolism; green, transmembrane proteins; and black, mobile elements. pAA^C700-09^ is compared with the five pAA plasmids; 55989, 042, 226, O86A1 and C227-11. Inner ring, GC content. The circular plasmid diagram was generated using BRIG.

BLAST comparison was performed on five completely sequenced pAA plasmids (**Table 3**) with pAA^C700-09^ (**Figure 3**). Although common features were observed among the pAA plasmids, many of which correspond to EAEC virulence genes, substantial variation in the genetic composition was observed, as previously described (Johnson and Nolan, 2009). Several virulence genes previously described on pAA plasmids were also encoded by pAA^C700-09^ including the cytotoxic SPATE protein Pet, the AggR transcriptional regulator, the AggR-activated regulator Aar, the surface protein dispersin and its secretion machinery Aat. Moreover, *senB* typically found on the Inv plasmid of *Shigella* and enteroinvasive *E. coli* encoding the shET2 toxin was located on the plasmid (Nataro et al., 1995b).

**Table 3:** Comparison of *E. coli* C700-09 to the other completely sequenced pAA plasmids.

*E. coli* Strain	*aggR*	*aap*	*aatA-P*	AAF variant	Toxins present on pAA	Other virulence factors	Size of pAA (basepairs)	Plasmid replicons	Accession number	Reference
C700-09	+	+	+	*agg3A, agg5A*	*pet, pic, senB*	*orf3, orf4, aar, capU, virK, shf, aaiA-aaiP*	120.286	IncFII, Col156	PRJEB18579	Boisen et al., 2012
55989	+	+	+	*agg3A*	*astA*	*orf3, orf4, aar*	72.482	IncFIB	NC_011752	Bernier et al., 2002
226	+	+	+	*agg5A*	*astA*	*orf3, orf4, aar*	102.786	IncFIB, IncFII	SRA055981	Dallman et al., 2012
042	+	+	+	*aafA*	*pet*	*orf3, orf4, aar capU, virK, shf*,	113.346	IncFIC	FN554767	Chaudhuri et al., 2010
C227-11	+	+	+	*aggA*	*–*	*orf3, orf4, aar*	74.217	IncFII, IncFIB	NC_018666	Scheutz et al., 2011
PO86A1	+	+	+	*agg4A*	*–*	*orf3, orf4, capU, virK, shf*,	120.730	IncII, IncFIB	AB255435	Johnson and Nolan, 2009

Like the other pAA plasmids, pAA^C700-09^ was a F-type plasmid with stability, maintenance and transfer regions. Whereas pAA^C700-09^ was of similar size as p042 and pO86A1, the F-transfer region was truncated, which was also observed in p55989 (Johnson and Nolan, 2009) indicating that pAA^C700-09^ is likely defective in self transfer ability. The pAA^C700-09^ shared the same replicon as the pAAs of the AAF/V expressing strain 226, the German outbreak strain C227-11 and O86A1 (IncFII), and not the pAA plasmid of 55989, which had the IncFIB replicon (**Table 3**).

Interestingly, using BLAST analysis, we found that unique regions of pAA^C700-09^ encoded several virulence genes typically located on the chromosome of EAEC or *Shigella* spp. isolates. These included a type VI secretion apparatus (Aai) normally found on the chromosome of EAEC which appears to have been inserted into the pAA plasmid via transposable elements. The *aai* cluster was first observed in strain 042, encoded on a 117 kb pathogenicity island inserted at the *pheU* tRNA site (Dudley et al., 2006). The prototype strain 042 was shown to harbor 26 genes in the aai cluster (*aaiA-aaiY*), whereas in pAA^C700-09^, the *aai* cluster comprised 16 genes *(aaiA-aaiP)* with 85% nucleotide identity to 042. Importantly, it has previously been shown that *aaiA-P* are sufficient for secretion of the effector protein AaiC (Dudley et al., 2006).

In addition to the *aai* cluster, the serine protease autotransporter Pic, normally located on the chromosome of EAEC and *Shigella* spp., was also located on pAA^C700-09^ (**Figure 3**) (Henderson et al., 1999; Behrens et al., 2002).

### The pAA^C700-09^ Plasmid is Found in Strains Positive for both AAF/III and AAF/V

**FIGURE 4:**
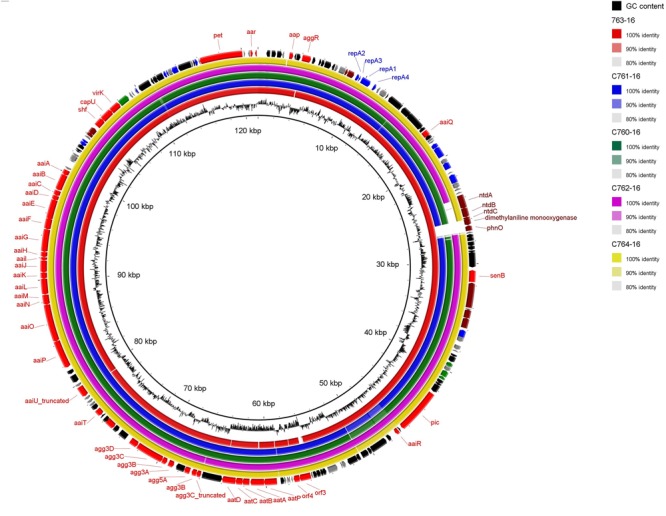
**Circular map of pAA^C700-09^ compared to the five other *agg3A+agg5A* expressing strains.** As shown in the BRIG alignment, pAA^C700-09^ is identical to the plasmids from strain C763-16, C761-16, C760-16, C762-16, and C764-16 except for a small region consisting of 3 kb encoding proteins responsible for catabolism. The outer ring shows predicted ORFs. Different colors represent different putative functions: gray, hypothetical proteins; red, EAEC-specific virulence factors; blue, plasmid replication and maintenance; maroon, catabolism and metabolism; green, transmembrane proteins; and black, mobile elements. Inner ring, GC content. The circular plasmid diagram was generated using BRIG.

**FIGURE 5:**
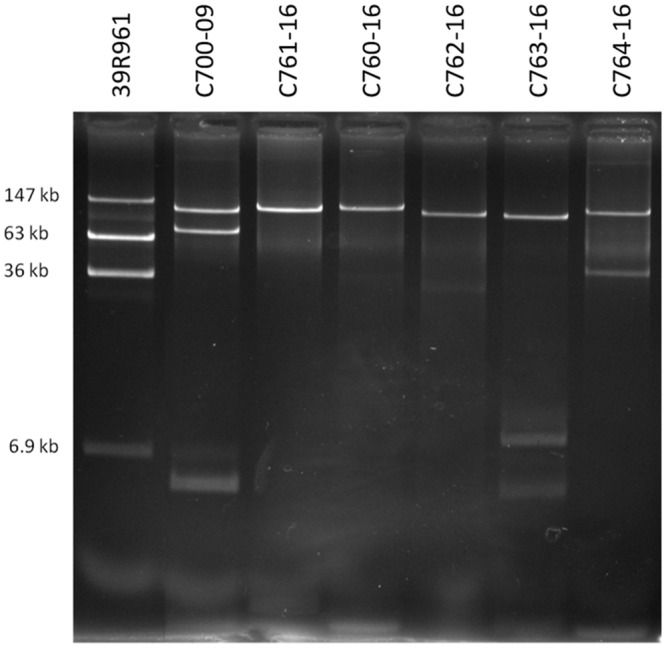
**Plasmid profiles of the six strains encoding *agg3A* and *agg5A.* Plasmid profiles with *E. coli* 39R861 as a marker (147, 63, 37, and 7 kb) in lane 1.** The plasmid profile showed that all of the *agg3A+agg5A* strains harbored a plasmid of approximately 120 kb.

We next performed Illumina-based whole-genome sequencing of the five additional EAEC strains harboring *agg3A+agg5A* in order to establish if they harbor plasmids similar to that of C700-09. BLAST analysis clearly indicated that all strains harbored the same pAA plasmid as C700-09 (**Figure 4**), except for a small region consisting of approximately 3 kb which contained genes encoding proteins involved in kanosamine synthesis pathway (*ntdABC*; Vetter et al., 2013) as well as *phnO* encoding an aminoalkylphosphonate N-acetyltransferase (Hove-Jensen et al., 2012). This was further verified by plasmid profiling of the six strains. Whereas the pAA plasmid of 55989 consists of 72 kb, the six strains all harbored a large plasmid of approximately 120 kb, verifying that all six strains harbor pAA^C700-09^ (**Figure 5**).

### Clonality of Strains Harboring pAA^C700-09^

**FIGURE 6:**
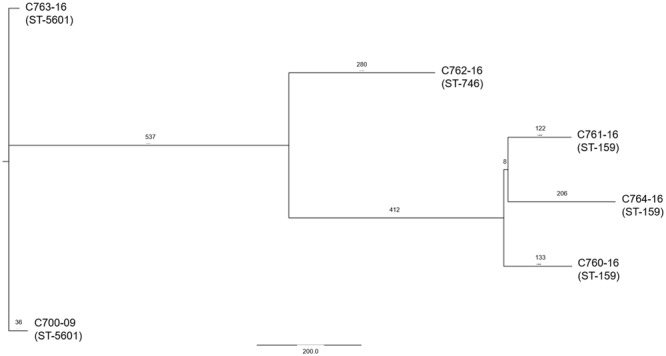
**A phylogenetic SNP tree of the six strains encoding *agg3A+agg5A*.** The strains were clustered completely according to their MLST type. The tree was reconstructed using an in-house pipeline and the SNPs were extracted from the six genomes. The numbers above the nodes corresponds to the number of SNPs present between the strains.

We next investigated the phylogenetic relations of the six strains harboring pAA^C700-09^. EAEC strains are known for their heterogeneity, and comprise many different serotypes as well as MLST types. The serotypes of the six strains were examined both *in silico* using the CGE pipeline^4^ as well as phenotypically and all six strains were found to belong to the same serotype O181:H19. MLST analysis using the CGE pipeline revealed that the strains belonged to three MLST types. However, these MLST types are single locus variants at the *adk* locus and therefore belong to the same MLST complex (Wirth et al., 2006). To further elucidate the phylogenetic relationship, a single-nucleotide polymorphism (SNP)-based phylogenetic tree was constructed. As expected, the isolates clustered according to their respective MLST type into three clusters. Importantly, although the six strains all belonged to the same MLST subgroup, the SNP analysis clearly revealed that pAA^C700-09^ is present in different strains (**Figure 6**).

### Both *agg3A* and *agg5A* Fimbrial Subunit Genes Are Expressed from pAA^C700-09^

**FIGURE 7:**
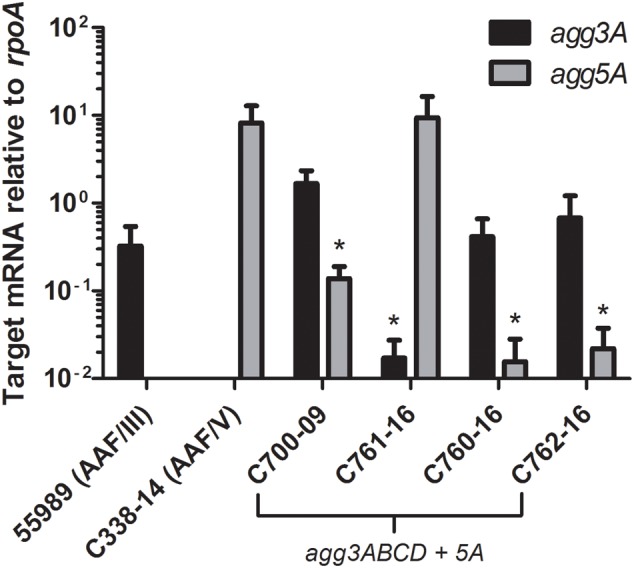
**qRT-PCR of four of the strains encoding *agg3A+agg5A* compared to the AAF/III and AAF/V wild type prototype strains.** Transcript levels for *agg3A* (black) and *agg5A* (gray) were evaluated in the prototype AAF/III-expressing strain 55989, the prototype AAF/V-expressing strain C338-14 and four strains harboring both *agg3*A and *agg5A* genes by qRT-PCR. Transcripts were examined during mid-log phase and results are normalized to the constitutively expressed housekeeping gene *rpoA*. Each value used for statistical analyses is the mean of three independent experiments. Results were considered significant when *P* < 0.05 (^∗^), *P* < 0.01 (^∗∗^), *P* < 0.001 (^∗∗∗^).

To investigate if both *agg3A and agg5A* fimbrial subunit genes were expressed, mRNA was isolated from four strains C700-09, C761-16, C760-16 and C762-16 and grown to mid log-phase and cultivated under AAF-inducing conditions in DMEM/0.5 % glucose (Morin et al., 2010) followed by RT-qPCR analysis. Interestingly, both fimbrial subunits genes were found to be transcribed in all four strains (**Figure 7**). Although the strains were grown in the exact same conditions, variations were observed between the patterns of *agg3A/5A* mRNA expression between the four strains. Three of the strains (C700-09, C760-16 and C762-16) had similar expression patterns, expressing significantly more *agg3A* mRNA compared to *agg5A* (*P* < 0.05), whereas the pattern was completely the opposite for strain C761-16 expressing *agg5A* at the same level as the wild type AAF/V strain C338-14, and with almost no *agg3A expression* (*P* < 0.05).

## Discussion

Enteroaggregative *E. coli* is an important diarrheal pathogen but its diagnosis and identification remains challenging due to the heterogeneity of the strains. Adherence to the intestinal mucosa is a crucial part of the pathogenesis, and is facilitated by AAFs. Most recently, a novel member of the AAF family was identified, thus bringing the number of AAF variants up to a total of five (Jonsson et al., 2015).

In order to detect all AAF variants, we optimized a previously described AAF multiplex PCR assay and screened a Danish collection of 162 clinical EAEC isolates. By use of the optimized assay, 87% of the isolates tested positive for an AAF variant, which is a significant improvement compared to previous studies, which only detected the presence of an AAF variant in approximately 50% of EAEC strains tested (Boisen et al., 2012; Lima et al., 2013). In general, we observed the same frequencies as previously described (Jonsson et al., 2015).

Interestingly, we found that 3% of the strains harbored genes encoding two of the pilin subunits *agg3A* and *agg5A*. These were revealed to be encoded by a novel pAA plasmid, pAA^C700-09^, harboring a complete AAF/III gene-cluster as well as a truncated AAF/V gene-cluster. Notably, it was previously shown that AAF/V and AAF/III share the genes encoding for *agg3DCB*, whereas the major pilin subunits share only 32.2% identity (Jonsson et al., 2015).

Although EAEC pAA plasmids share a common plasmid backbone and core EAEC-associated virulence genes, the genetic compositions of the pAA plasmids of prototype EAEC strains are quite different from each other (Johnson and Nolan, 2009). Our study supports this, as pAA^C700-09^ contains several virulence genes not found on the previously completely sequenced pAA plasmids. This includes a variant of the Aai cluster encoding a type VI secretion system as well as the gene encoding the Pic toxin, which is typically chromosomally encoded in EAEC. Thus, acquisition of this plasmid endow the host strain with a remarkably high number of EAEC associated virulence genes hereby, likely promoting strain pathogenicity.

The population structure of EAEC is seen to be very heterogeneous (Okeke et al., 2010; Chattaway et al., 2014). Whole-genome SNP analysis of the six strains harboring pAA^C700-09^, revealed that although the strains belonged to three related MLST types, pAA^C700-09^ is found in clearly distinct strains. The phylogenetic resemblance of the strains may reflect that the plasmid was first obtained by a common ancestor, and has then due to fitness advantages been maintained in this clonal lineage. It is epidemiological interesting to clarify whether this plasmid is present in other clonal lineages.

Lastly, we wanted to investigate whether or not both variants were expressed. The levels of *agg3A* and *agg5A* mRNA expression was therefore quantified in four strains revealing that *agg5A* as well as *agg3A* were transcribed in all of the strains. Importantly, as AAF/III and AFF/V share accessory genes, it is likely that the Agg5A protein is assembled into functional fimbriae via expression of the accessory genes from the intact AFF/III gene cluster. Notably, variations were observed between the expression patterns between the four strains harboring pAA^C700-09^. This could be related to the different genetic background of the strains but further studies are needed to clarify this.

The study provides valuable new information regarding the complexity of EAEC pathogenicity and the discovery of a novel pAA plasmid encoding a wide array of EAEC virulence genes and potentially enabling expression of two AAF variants are intriguing. However, several aspects remain to be elucidated. Although we establish that the genes encoding both types of major pilin subunits are transcribed, the fimbrial expression on the bacterial surface remains to be verified and characterized. Thus, further studies are needed to establish whether the fimbriae are expressed, and if so, whether they are composed of chimeric subunits of Agg3A and Agg5A polymerized into one long fimbria or two separate fimbriae. It would possibly be advantageous for the bacteria to express two separate fimbriae, as it would, not only enable binding to alternative host receptors but also potentially make the bacteria capable of exploiting different niches in the human gut. Additionally, AAFs have also previously been shown to cause inflammatory responses (Harrington et al., 2005; Boll et al., 2012), thereby being easily recognized by the host. Thus, the ability to switch between the expression of two fimbriae variants could be speculated to restrain the host immune response. Furthermore, as the two fimbriae variants have different binding specificities the individual variants could be more favorable to express in certain environments. This relationship has been shown for uropathogenic *E. coli* strains and their ability to switch between type 1 fimbriae and P-fimbriae expression when colonizing the lower and upper urinary tract, respectively (Melican et al., 2011).

More studies are needed to establish how the fimbriae are composed, the effect on pathogenicity as well as the impact of the additional genes inserted on this novel pAA plasmid.

## Author Contributions

KK and HJ obtained the funding and were responsible for the project. BJ collected the bacterial isolates used in the study. RJ, CS, CAS, and EB were responsible for the microbiological analyses performed in the study. FS, KJ, and NB were responsible for the serotyping and SNP analysis. RJ, CS, NB, EB, HJ, and KK were responsible for the interpretation of the research data and of the microbiological analysis. All authors have contributed to the writing of the manuscript and have all approved the final draft of the manuscript. All authors take responsibility for the accuracy and integrity of the research performed.

## Conflict of Interest Statement

The authors declare that the research was conducted in the absence of any commercial or financial relationships that could be construed as a potential conflict of interest.

## Footnotes

1 https://www.ebi.ac.uk/ena

2 https://www.ebi.ac.uk/ena

3 https://cge.cbs.dtu.dk/services/SerotypeFinder/

4 https://cge.cbs.dtu.dk/services/
